# Focal cavity radiotherapy after neurosurgical resection of brain metastases: sparing neurotoxicity without compromising locoregional control

**DOI:** 10.1007/s00066-022-02003-3

**Published:** 2022-09-23

**Authors:** Klaus-Henning Kahl, Ehab Shiban, Susanne Gutser, Christoph J. Maurer, Björn Sommer, Heiko Müller, Ina Konietzko, Ute Grossert, Ansgar Berlis, Tilman Janzen, Georg Stüben

**Affiliations:** 1 Klink für Strahlentherapie und Radioonkologie, Universitätsklinikum Augsburg, Stenglinstr. 2, 86256 Augsburg, Germany; 2 Klinik für Neurochirurgie, Universitätsklinikum Augsburg, Augsburg, Germany; 3 Medizinische Physik und Strahlenschutz, Universitätsklinikum Augsburg, Augsburg, Germany; 4 Klinik für Diagnostische und Interventionelle Radiologie und Neuroradiologie, Universitätsklinikum Augsburg, Augsburg, Germany; 5 Interdisziplinäres Zentrum für Palliative Versorgung, Universitätsklinikum Augsburg, Augsburg, Germany

**Keywords:** SRS, Whole brain radiotherapy, WBRT, Radionecrosis, Leptomeningeal disease

## Abstract

**Purpose:**

Does focal cavity radiotherapy after resection of brain metastasis “spare” whole-brain radiotherapy, which is associated with toxicity for patients, through the complete course of their disease without compromising long-term local control of the brain?

**Methods:**

We retrospectively analyzed outcomes of patients who underwent adjuvant focal cavity radiotherapy between 2014 and 2021 at our center.

**Results:**

A total of 83 patients with 86 resected brain metastases were analyzed. 64% had singular, 36% two to four brain metastases. In cases with multiple metastases, omitted lesions were treated with radiosurgery. Median follow-up was 7.3 months (range 0–71.2 months), 1‑year overall survival rate was 57.8% (95% CI 44.9–68.8%). Radiotherapy was administered with a median biologically effective dose (α/β 10) surrounding the planning target volume of 48 Gy (range 23.4–60 Gy). Estimated 1‑year local control rate was 82.7% (95% CI 67.7–91.2%), estimated 1‑year distant brain control rate was 55.7% (95% CI 40.5–68.4%), estimated 1‑year leptomeningeal disease rate was 16.0% (95% CI 7.3–32.9%). Eleven distant brain recurrences could be salvaged with radiosurgery. In the further course of disease, 14 patients (17%) developed disseminated metastatic disease in the brain. Estimated 1‑year free of whole-brain radiotherapy rate was 72.3% (95% CI 57.1–82.9%). All applied treatments led to an estimated 1‑year neuro-control rate of 79.1% (95% CI 65.0–88.0%), estimated 1‑year radionecrosis rate was 23% (95% CI 12.4–40.5%).

**Conclusion:**

In our single-center study, focal cavity radiotherapy was associated with high local control. In three out of four patients, whole-brain radiotherapy could be avoided in the complete course of disease, using radiosurgery as salvage approach without compromising neuro-control.

## Introduction

Neurosurgical resection is standard of care for patients suffering from large symptomatic brain metastases [1]. Even in highly selected cases, surgical resection alone leads to 1‑year local control rates of only around 40% [2]. Since the 1990s, postoperative radiotherapy has become the gold standard for these patients. Improvement of systemic oncological therapies has meanwhile led to prolonged survival of metastatic cancer patients. Therefore, long-term neurotoxicity resulting from former standard whole-brain radiotherapy becomes more and more relevant and affects patients’ functional outcome. Randomized trials have shown the detrimental effect of whole-brain radiotherapy on neurocognitive functioning [3]. This led to the treatment strategy of focal cavity radiotherapy following surgery [4, 5]. However, this treatment strategy cannot address the risk of tumor recurring in the brain distant from the resection cavity, and coping systemic treatment options are lacking currently. At Augsburg University Medical Center (UKA), the interdisciplinary neuro-oncology board established a treatment strategy in 2014 composed of regular 3‑monthly MRI follow-up and radiosurgery as the basis for salvage treatment of distant “oligo-”metastatic brain failure.

This study presents retrospective data on our strategy to spare whole-brain radiotherapy, which is known to be associated with toxicity for patients, through the complete course of their disease. Furthermore, it provides data on long-term local control of the brain (“neuro-control”).

## Methods

### Data collection

We scanned the data from our hospital information system ORBIS (Dedalus Healthcare Group GmbH, Bonn, Germany), our radiology information system Deep Unity (Dedalus Healthcare Group GmbH, Bonn, Germany), and our oncology information system MOSAIQ (ELEKTA AB, Stockholm, Sweden) for patients who underwent neurosurgical resection of brain metastases followed by focal percutaneous radiotherapy of the resection cavity and who did not receive whole-brain radiotherapy. Treatments took place between 2014 and 2021 at UKA. The time point for the last follow-up included was December 31, 2021.

### Treatment

Treatment of all cases followed the recommendations of the UKA multidisciplinary tumor board. After obtaining informed consent from the patient, neurosurgical brain metastasectomy was performed. Within 72h after surgery, a postoperative MRI was performed to evaluate resection status.

### Radiotherapy treatment planning

For radiotherapy treatment planning, all patients were immobilized with an individually customized thermoplastic mask in supine position and scanned with a Somatom Confidence CT (Siemens Healthineers AG, Erlangen, Germany). The dataset was reconstructed in 1‑mm slices. An additional MRI with intravenous gadolinium was obtained on the same or, at the latest, the following day, and reconstructed in 1‑mm slices for image fusion. Board-certified radiation oncologists performed target volume definition and contouring. The gross tumor volume (GTV) was defined as the resection cavity including the contrast-enhancing rim/suspected residual tumor. For all treatments, a clinical target volume (CTV) was created by a 1-mm expansion of the GTV within surrounding brain or meningeal tissue. As the standard neurosurgical resection procedure of brain metastases with meningeal contact in our center includes wide meningeal resection, no additional margins along the meninges were added in these cases. Planning target volume (PTV) was defined as CTV plus 2 mm.

In 2014, our institutional policy changed. With increasing data regarding the advantages of fractioned stereotactic radiotherapy, we moved away from single-fraction treatment in cavity treatment. However, in two patients in this cohort, a single radiation dose of 20 Gy was prescribed to the 80% isodose surrounding the PTV margin, demanding that at least 98% of the PTV should receive 100% of the prescribed dose.

For the vast majority of patients, a radiation dose of five fractions of 6 Gy was prescribed to the PTV margin, demanding that at least 98% of the PTV should receive 100% of the prescribed dose. In case of suspected residual disease in the postoperative MRI [6], the radiation dose was escalated to five fractions of 7 Gy. For two patients with large cavities, the prescribed dose/fractionation had to be changed to 13 × 3 Gy to meet the dose constrains of organs at risk. One of these patients only received 6 × 3 Gy due to a severe pulmonary infection, requiring intensive care unit treatment.

### Radiation treatment

Radiotherapy was performed as frameless, image-guided stereotactic radiotherapy with a True Beam linear accelerator (Varian Medical Systems, Palo Alto, USA). Radiotherapy fractions were given on consecutive working days.

### Follow-up

After treatment, all patients received standardized follow-up (FU) including 3‑monthly MRI of the brain according to UKA FU policy after focal brain radiotherapy. Without exceptions, all decisions for further treatments followed the recommendations of the UKA multidisciplinary tumor board.

### Neuroradiological assessment

Assessment of FU MRIs followed the response assessment in neuro-oncology (RANO) criteria for brain metastases [7].

In the case of a suspected local relapse, MRI perfusion [8] and PET scan with F‑18-fluorethyltyrosin (FET) were used for non-invasive distinction between tumor relapse and radionecrosis. In case of persistent suspicion of local tumor regrowth, neurosurgical resection of the lesion was recommended to the patient.

### Statistical analyses

All statistical analyses were performed with EZR (version 3.4.1/the R Foundation for Statistical Computing, Vienna, Austria) [9] using Kaplan–Meier methods, Cox proportional hazard regression, and log-rank tests.

## Results

We analyzed the course of 83 patients with a median age of 63 years (range 34–87 years) with 86 resected brain metastases. Fifty-three patients (64%) had a singular brain metastasis, 16 patients (19%) two brain metastases, 12 patients (14%) three, and two patients (2%) four brain metastases at the time of treatment. Three of these metastases were resected; all others were treated with radiosurgery only. At the time of analysis, 36 of all patients had died, 34 were alive, and the status of 13 patients was unknown. Twenty-five of the deceased patients (75%) had no radiologic signs of central nervous system progression on their last cerebral MRI. Main causes of death were systemic progressive disease and infections. Median follow-up and MRI follow-up after radiotherapy was 7.3 months (range 0–71.2 months) and 6.4 months (range 0–64.3 months). Estimated 1‑year overall survival rate and median survival were 57.8% (95% CI 44.9–68.8%) and 1.5 years (95% CI 0.8 year–not applicable (NA)), respectively. Table 1 provides additional patient characteristics.

**Table 1:** Patient, disease, and treatment characteristics

*Age*	Median	63 years	Range: 34–87 years
*Sex*	Female	48 patients	58%
	Male	35 patients	42%
*RPA*	Class 1	12 patients	15%
	Class 2	55 patients	66%
	Class 3	16 patients	19%
*Systemic tumor burden*	None other than brain	23 patients	28%
	Additional tumor sites	60 patients	72%
	Only 1 organ	13 patients	–
*Number of brain metastases per patient*	Median	1 brain metastasis	Range: 1–4 metastases
	1 brain metastasis	53 patients	64%
	2 brain metastases	16 patients	19%
	3 brain metastases	12 patients	14%
	4 brain metastases	2 patients	2%
*Localization of brain metastases*	Frontal	13 metastases	15%
	Occipital	14 metastases	16%
	Parietal	18 metastases	21%
	Temporal	15 metastases	17%
	Posterior fossa	26 metastases	30%
*Brain radiotherapy history*	No previous brain RT	74 patients	90%
	Previous brain RT	8 patients	10%
*Size of resected metastasis*	Median	34 mm	Range: 10–80 mm
	Mean	34 mm	–
	1–10 mm	4 metastases	5%
	10–20 mm	10 metastases	12%
	20–30 mm	22 metastases	26%
	30 mm +	49 metastases	58%
*OR time per patient*	Median	139 minutes	Range: 52–254 minutes
*Suspected incomplete resection on postop MRI*		29 lesions	34%
*Histology*	Lung cancer: – Adenocarcinoma – Other NSCLC histology	25 20 (1 ALK/8 KRAS mut.) 5 (0 PD-L1 > 30%)	29%
	Breast cancer	20 (16 Her2-neu+)	23%
	Malignant melanoma	14 (11 BRAF mut.)	15%
	Colorectal cancer	9 (4 KRAS mut.)	10%
	Ovarian cancer	3 (1 BRCA mut.)	3%
	Gastroesophageal cancer	3 (3 MSS/Her2-neu −)	3%
	Renal cancer	2	2%
	Misc.^a^	7	8%
*Time from surgery to discharge*	Median	7 days	Range: 3–41 days
*Interval between surgery and start of radiotherapy*	Median	29 days	Range: 14–78 days
*BED (a/ß* *=* *10) at PTV margin*	Median	48 Gy	Range: 23.4–60 Gy
*Number of radiotherapy fractions*	Median	5 fractions	Range: 1–13 fractions
*Duration of radiotherapy course*	Median	6 days	Range: 1–19 days
*Follow-up* (Status on 31.12.2021)	Median	7.3 months	Range: 0–71.2 months
	Dead	36 patients	43%
	Alive	34 patients	41%
	Lost to follow up	13 patients	16%
*Follow up (MRI)*	Median	6.4 months	Range: 0–64.3 months
	Mean	13.3 months	–

*BED* biological effective dose, *CUP* cancer of unknown primary, *MRI* magnetic resonance imaging, *OR* operation room, *PTV* planning target volume, *RT* radiotherapy

^a^Fallopian tube, CUP, prostate, bladder, larynx, corpus uteri, cervix uteri

Postoperative MRI report suspected incomplete resection of 29 lesions (34%). Median interval between brain surgery and start of radiotherapy was 29 days (range 14–78 days). External beam radiotherapy was administered with a median of five fractions (range 1–13 fractions) over a median period of 6 days (range 1–19 days) to a median biological effective dose (α/β 10) of 48 Gy (range 23.4–60 Gy) at the margin of the planning target volume. Ten patients underwent salvage surgery due to suspected regrown tumor in the resection cavity (i.e., local failure) during follow-up in MRI ± FET-PET. Only in five of these cases did histology reveal vital tumor cells.

**Fig. 1:**
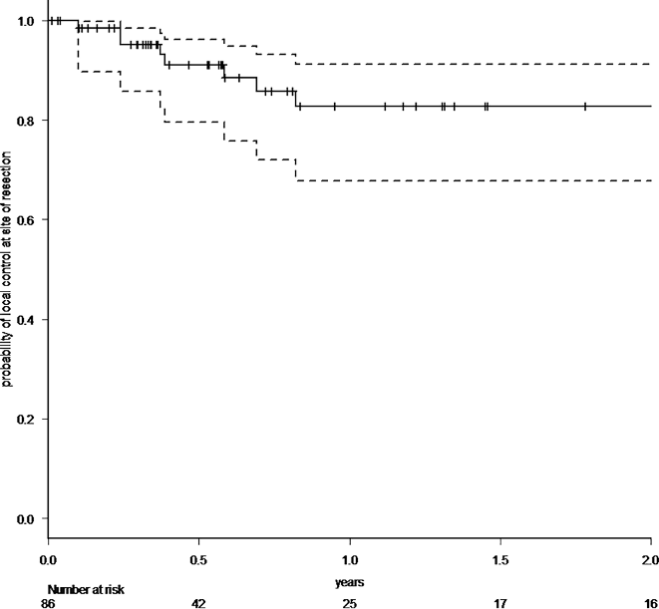
Probability of local control at the initial tumor site after resection of brain metastases and focal radiotherapy (the *dotted lines* represent the 95% confidence intervals)

**Fig. 2:**
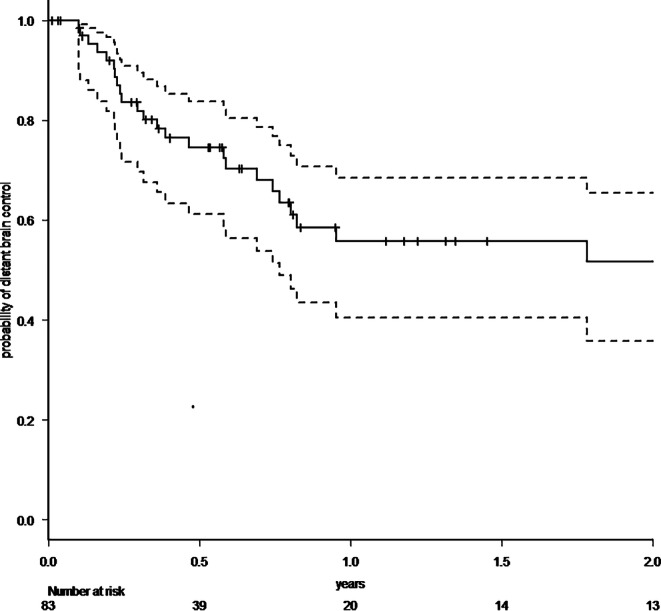
Probability of distant brain control after resection of brain metastases and focal radiotherapy (the *dotted lines* represent the 95% confidence intervals)

**Fig. 3:**
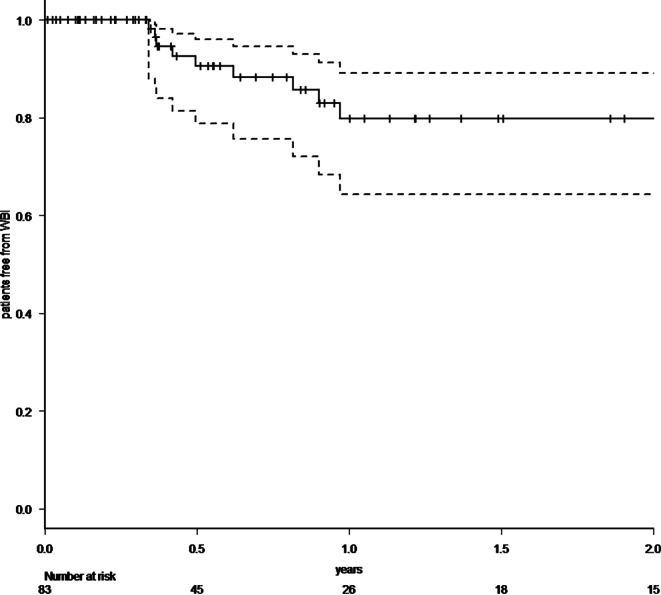
Probability of being free from whole-brain radiotherapy (WBI) after resection of brain metastases and focal radiotherapy (the *dotted lines* represent the 95% confidence intervals)

**Fig. 4:**
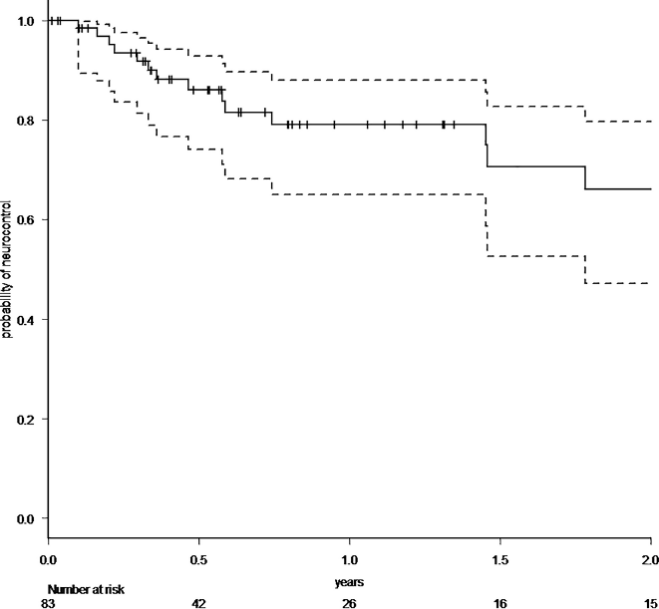
Probability of neuro-control (= no progressive disease in the brain on the last obtained MRI) after initial resection of brain metastases with sequential focal radiotherapy and all following salvage treatments (the *dotted lines* represent the 95% confidence intervals)

Within our standardized follow-up, we detected eight local recurrences (9.3%) in total. The 1‑year local control rate at the resection site was estimated at 82.7% (95% CI 67.7–91.2%; Fig. 1). There was no statistically significant difference in local control at the resection site between patients with complete and suspected incomplete resection. In 25 patients (30.1%), recurrences occurred in distant areas of the brain, leading to an estimated 1‑year distant brain control rate of 55.7% (95% CI 40.5–68.4%) (Fig. 2). Within the subset of these distant relapses, six cases of leptomeningeal disease (7.2%) were observed. Estimated 1‑year leptomeningeal disease rate was 16.0% (95% CI 7.3–32.9%). Eleven distant brain recurrences could be salvaged with radiosurgery. During the whole course of disease, 14 patients (17%) developed disseminated metastatic disease in the brain, thus demanding whole-brain radiotherapy (WBI). In all these patients, this was the first manifestation of a distant recurrence after focal cavity radiotherapy. Median time between the end of WBI and death was 65 days (9–1232 days). Kaplan–Meier survival analysis showed that 72.3% of all patients (95% CI 57.1–82.9%) were estimated to be free of WBI after 1 year (Fig. 3). The estimated 1‑year WBI-free survival rate was 51.3% (95% CI 38.7–62.5%). In summary the course of all applied treatments led to an estimated 1‑year neuro-control rate of 79.1% (95% CI 65.0–88.0%) in our set of patients after focal cavity radiotherapy with controlled disease in the brain on the last obtained MRI scan (Fig. 4). Five patients (6%) died within 30 days after completion of radiotherapy, mostly due to infections. No radiotherapy-associated cause of death was observed. After brain metastasis resection, 56 patients (67%) received palliative systemic treatment, which started at a median of 54 days (range 9–290 days) after surgery. Fourteen patients received immune checkpoint inhibitors (17%), 13 patients Her2-neu-targeted antibodies (16%), 17 patients cytotoxic chemotherapy (20%), nine patients targeted therapeutic agents (11%), four patients VEGF-directed antibodies (5%), and two patients anti-hormonal therapy as single-agent or combined therapy. In univariate analysis, systemic treatment, type of systemic treatment, histology, localization, size of metastasis, controlled systemic disease, and interval to start of radiotherapy had no impact on the development of recurrences in distant parts of the brain or leptomeningeal disease. The number of brain metastases showed a hazard ratio of 1.487 (95% CI 1.001–2.197; *p* = 0.04619) for distant brain failure in a Cox proportional hazards regression model. During their follow-up, 18 patients (21%) developed signs of radiotherapy-associated contrast-enhancing brain lesions (radionecrosis) on MRI scans, predominantly without any neurologic symptoms. Estimated 1‑year radionecrosis rate was 23% (95% CI 12.4–40.5%). Symptomatic radionecrosis occurred in five patients (6%), leading to estimation of a 1-year symptomatic radionecrosis rate of 3.6% (95% CI 0.5–22.8%). All symptomatic radionecrosis patients received a treatment of four infusions of bevacizumab (7.5 mg/kg every 2 weeks), which led to a significant improvement of their neurologic symptoms.

## Discussion

The findings of our retrospective analysis showed that focal radiotherapy of the cavity after resection of brain metastases was associated with high local control rates. Furthermore, it could be shown that whole-brain radiotherapy could be avoided for three out of four of our patients in the complete further course of their disease, using radiosurgery to salvage observed distant brain relapse.

Our treatment strategy was associated with a high definitive neuro-control rate of 75% for all patients. This was in line with the outcome of the NCCTG N107C/CEC.3 trial showing no difference in overall survival of patients treated with focal radiotherapy versus patients treated with whole-brain radiotherapy in this situation [3]. The reported local control rate in this randomized study (61.8%) was remarkably lower than the one we found in our retrospective analysis. Possible explanations for this fact could be differences in target volume and dose concepts, as well as in the limitations of a monocentric retrospective analysis. Our observed local control rate was nevertheless in the range between 80 and 90% reported by other centers [5, 10–12].

As expected from published data [13–15], distant brain failure after focal radiotherapy of the resection cavity was found frequently in our analysis, stressing the importance of continuous follow-up including MRI. Leptomeningeal disease was found in 7.2% of the cases in our study. This is in line with published data [10, 16, 17]. Nearly half of the distant brain recurrences could be salvaged with radiosurgery. This contributed to the high estimated 1‑year WBI-free rate of 72.3% (95% CI 57.1–82.9%) and the simultaneously high estimated 1‑year neuro-control rate of 79.1% (95% CI 65.0–88.0%) of our retrospective analysis. In our dataset, no evidence for an additional benefit in terms of locoregional control in the brain could be shown for systemic treatments. This was supported by a subset analysis, which gave no evidence of an effect of type of systemic treatment, interval to start of systemic treatment after resection, or controlled systemic disease status of the patient. The heterogeneity and limited number of patients in our study might bias this.

Radiotherapy-associated contrast-enhancing brain lesions, i.e., radionecrosis [18, 19], have been inconsistently reported in publications so far. This is due to the fact that the differential diagnosis to local progression is challenging. In our series, those MRI findings were detected in 21% of all patients during follow-up. Most were diagnosed in asymptomatic patients and regressing or stable without any further treatment. Five of them were histologically proven by complete resection of the lesion due to suspected local recurrence on MRI ± FET-PET. The estimated 1‑year radionecrosis rate of 23% was in the range of other reported series [4, 5], while the 1‑year symptomatic radionecrosis rate of 3.6% was quite low in the light of these data.

The findings of this analysis have all the limitations of a retrospective study, which is liable to selection bias and incomplete follow-up data. Its limited sample size did not allow exploring of certain covariates like the influence of adjuvant treatment or specific genetic mutations on long-term neuro-control. For this purpose, larger multi-institutional register studies would be desirable.

## Conclusion

In our study, focal radiotherapy of the tumor cavity, replacing whole-brain radiotherapy after resection of brain metastases, was associated with high local control. For three out of four patients, whole-brain radiotherapy could be avoided in the complete further course of their disease, using radiosurgery to salvage observed distant brain relapse without compromising neuro-control.
